# From classic models to new pathways: unraveling the anatomy and function of the inferior fronto-occipital fasciculus in language processing

**DOI:** 10.3389/fpsyg.2025.1561482

**Published:** 2025-04-02

**Authors:** Pedro Aleixo Nogueira, Julia Franco Neiva, Maíra Piani Couto, Marcus Vinicius Giglio, Marcos Vinicius Calfat Maldaun, Andrei Fernandes Joaquim, Enrico Ghizoni, Cleiton Formentin

**Affiliations:** ^1^Department of Neurology, State University of Campinas, Campinas, Brazil; ^2^Department of Neuroscience, Hospital Sirio Libanes, São Paulo, Brazil

**Keywords:** inferior fronto-occipital fasciculus, language, semantic, cognitive function, neurosurgical planning, glioma surgery

## Abstract

**Introduction:**

This study explores the anatomy and function of the inferior fronto-occipital fasciculus (IFOF), focusing on its role in language processing. Through a comprehensive systematic review and detailed anatomical dissections, we aim to elucidate the IFOF’s anatomical organization, its contributions to language processing, and its complex three-dimensional configuration, ultimately enhancing the safety and precision of neurosurgical practices.

**Methods:**

This study employed a two-part methodology: (1) anatomical dissections using Klinger’s technique on three human brains, which were fixed and frozen; and (2) a systematic literature review adhering to PRISMA guidelines, with a search of the EMBASE and PubMed databases on January 1, 2025, analyzing 510 studies on IFOF anatomy and function, with a focus on its role in language processing and implications for neurosurgical practice.

**Results:**

Anatomical dissections identified the IFOF as a prominent anterior–posterior white matter tract with distinct dorsal and ventral components. The dorsal component links the pars triangularis and pars orbitalis of the frontal lobe to the superior parietal lobe and posterior occipital gyri, while the ventral component connects the inferior occipital gyrus and posterior basal temporal region to the dorsolateral prefrontal and orbitofrontal cortices. The IFOF was found to traverse through key areas, including the extreme capsule, insula, and claustrum, and was closely associated with the uncinate fasciculus. The systematic literature review included 15 studies, highlighting the IFOF’s critical role in cognitive and linguistic functions, particularly in semantic language processing, reading, naming, and integrating visual information for meaning interpretation. It plays a key role in language comprehension by connecting posterior visual regions to anterior semantic areas. The IFOF also contributes to visual attention and spatial processing, underscoring its importance in contemporary linguistic models. Damage to the IFOF can cause semantic paraphasia, reading difficulties, spatial neglect, and aphasia, highlighting its crucial role in language and cognitive functioning.

**Conclusion:**

The IFOF plays a pivotal role in integrating visual, motor, and semantic information, facilitating complex interactions between cognitive, linguistic, and visuospatial functions. Its dorsal component aids visuospatial integration, while the ventral component underpins semantic processing. The IFOF’s anatomical and functional complexity underscores its critical consideration in neurosurgical planning.

## 1 Introduction

For over a century, the classical model of language localization focused on the division between motor and sensory language areas, specifically Broca’s and Wernicke’s areas. This model was widely accepted as the cornerstone of language processing (Chang et al., 2015). However, advances in neuroimaging and brain stimulation have significantly reshaped our understanding, introducing a more dynamic model involving two primary pathways: the “dorsal” and “ventral” streams. The dorsal pathway, which connects the posterior superior temporal and inferior frontal cortices, is responsible for phonological processing. In contrast, the ventral pathway mediates semantic processing, running from the temporal pole to the basal occipitotemporal cortex and establishing anterior connections (Chang et al., 2015; de Benedictis et al., 2021; Shekari and Nozari, 2023; Dick et al., 2014; Mandonnet, 2017; Zhang et al., 2021; Bucheli et al., 2014; Motomura et al., 2014).

Cadaveric brain dissections, combined with advanced neuroimaging techniques such as tractography, have deepened our understanding of white matter tracts. These insights contribute to safer neurosurgical approaches, reducing postoperative complications and improving patient outcomes by preserving critical language pathways (Henderson et al., 2020; Vandermosten et al., 2012; Pescatori et al., 2017).

This study focuses on the anatomy and function of the inferior fronto-occipital fasciculus (IFOF), with a particular emphasis on its role in language processing and the clinical repercussions of its injury. It combines a systematic review of the IFOF’s functional significance with anatomical dissections to provide a comprehensive understanding of its structure. The objectives are to elucidate the anatomical organization, clarify its role in human language processing, and describe its complex three-dimensional architecture.

## 2 Methods

This methodological approach was based on two key components: a systematic literature review and precise brain dissection.

### 2.1 Anatomical dissections

**Figure 1:**
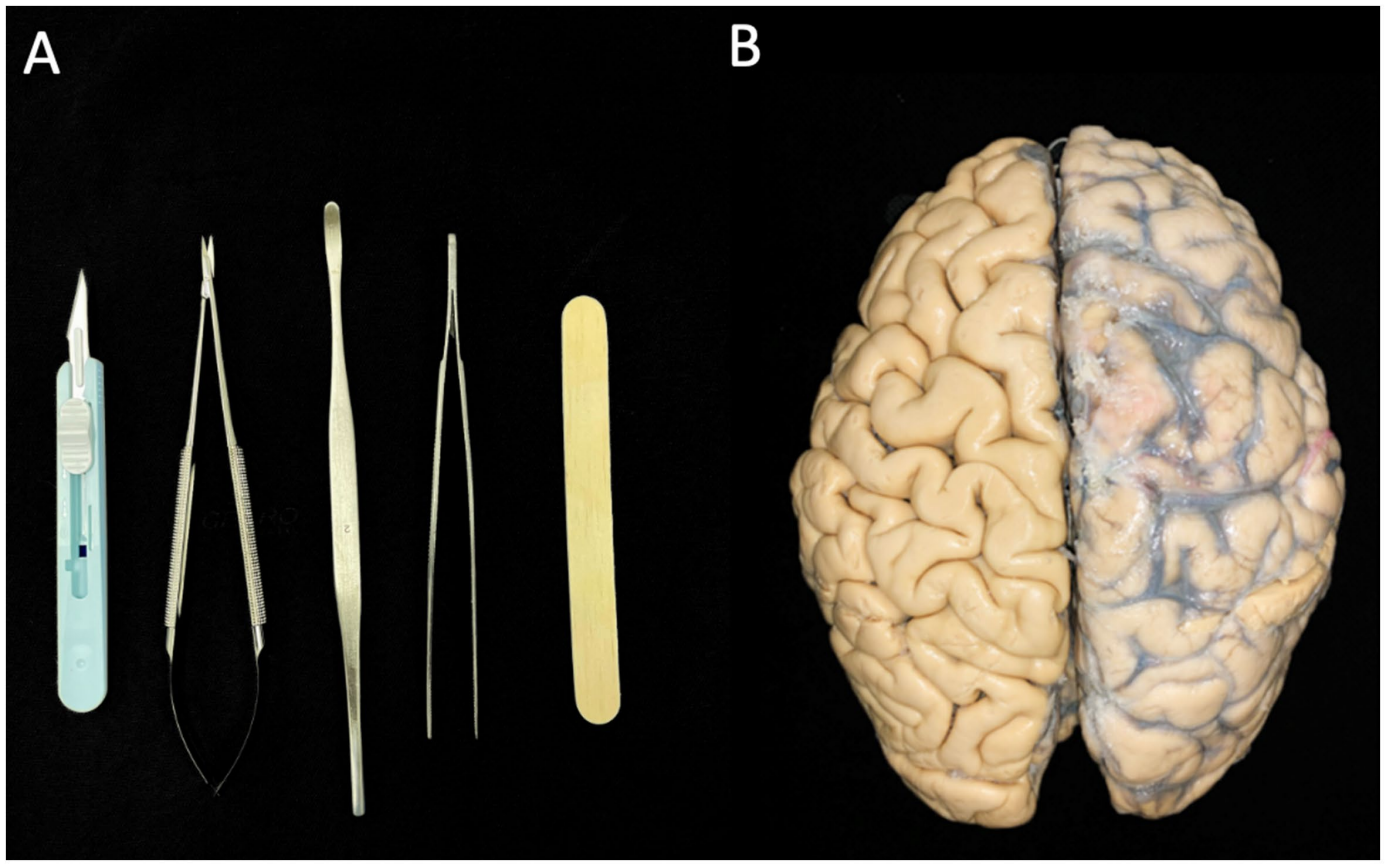
**(A)** Instruments used for dissecting the brain hemispheres. **(B)** Two intact brain hemispheres. The left hemisphere retains the vessels, arteries, and arachnoid membrane, while these structures have already been dissected in the right hemisphere.

Klinger’s technique was utilized to perform brain dissections on three human brains, which had been fixed in a solution of one-third alcohol and two-thirds water for a minimum of 30 days. The dissection process began with the removal of the arachnoid membrane and associated vascular structures (vessels and arteries). The hemispheres were subsequently frozen at −20°C for 2 weeks. Dissection primarily involved the use of wooden spatulas of varying sizes; however, precise dissection was conducted with scalpels, microsurgical scissors, a microsurgical clamp, and other dissecting instruments (Figure 1A). Before initiating the dissection, a thorough analysis was essential to understand the superficial anatomy of each brain lobe, enabling the identification of variations in gyral and sulcal patterns (Figure 1B). This step was crucial for correlating the cortical connections of the fasciculi in subsequent stages of dissection. To expose the white matter, we adopted a lateral-to-medial approach. Images of the dissection were captured using a Nikon D40 camera.

### 2.2 Literature review

This systematic review was conducted following the PRISMA (Preferred Reporting Items for Systematic Reviews and Meta-Analyses) guidelines. An online search was performed on January 1, 2025, using the terms “IFOF” OR “inferior fronto-occipital fasciculus” in the EMBASE and PubMed (MEDLINE) databases. Filters were applied to exclude case reports, abstracts, and posters, resulting in 510 studies, with 198 from MEDLINE and the remainder from EMBASE. Inclusion criteria were limited to studies published in English that explored the role and function of the IFOF, focusing on its anatomical structure and physiological contributions, particularly in language maintenance and development. To ensure thoroughness, only full-text articles meeting these criteria were included, facilitating a systematic and comprehensive review process.

## 3 Results

### 3.1 Anatomical dissections

**Figure 2:**
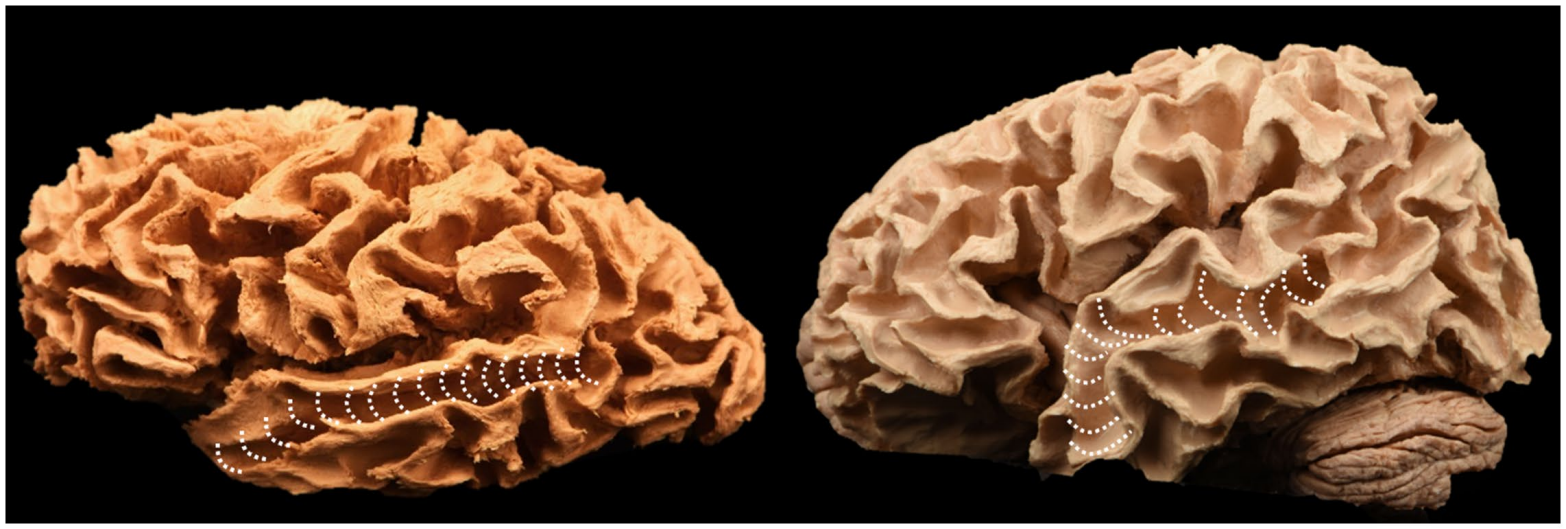
Short association fibers, also known as “U” fibers, exposed following cortex removal.

We dissected three human brains (six hemispheres – three right and three left) using Klinger’s technique and found no differences between the left and right sides. The cortical contents of the lateral brain surface were systematically removed, starting with the frontal lobe and proceeding through the parietal, temporal, and occipital lobes. Removing the cortical layer allowed visualization of the subcortical U-fibers, also known as short association fibers, responsible for the connections between adjacent gyri of the brain (Figure 2) (Martino and De Lucas, 2014).

**Figure 3:**
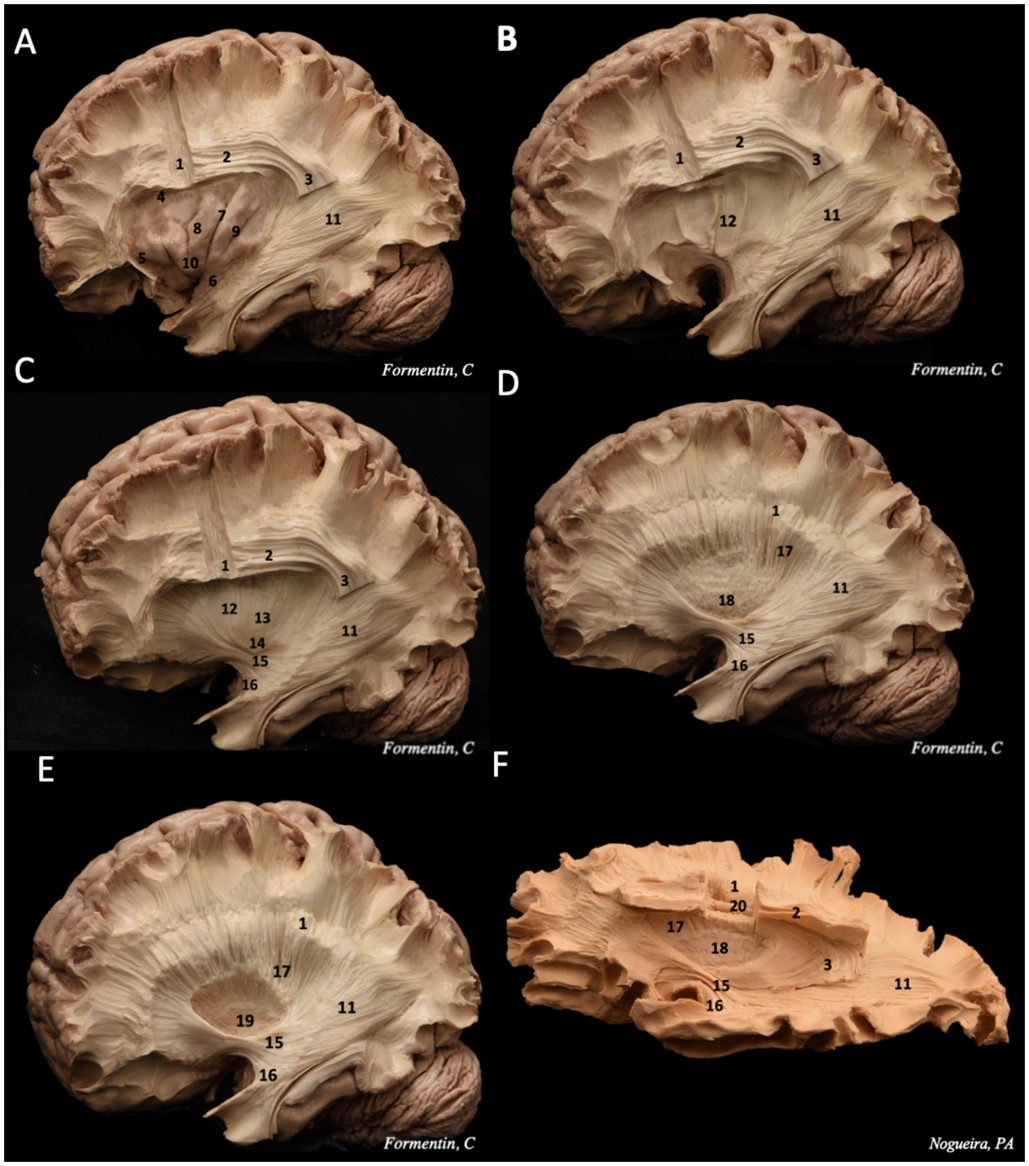
Step-by-step dissection of white fibers, progressing from the lateral to the medial surface. **(A)** We removed the frontal, temporal, parietal and occipital cortex, following by the removal of the short association fibers, remaining the insulae cortex intact. These steps allow to visualize the morphology of the superior longitudinal fasciculus, divided in horizontal and vertical portions, and the sagittal stratum, deeper in the posterior region. **(B)** exposure of the extreme capsule, a series of nerve tracts adjacent to the insulae cortex. **(C)** dissecting deeper into the central core, it is possible to identify in sequence the claustrum layer, and then the external capsule, also the IFOF and UF are seeing. **(D)** removing the external capsule, the putamen is exhibited. **(E)** then the globus pallidus, which both structures constitute the lentiform nucleus. **(F)** It is possible to observe the close relation between the corona radiata (superficial) and the lateral ventricle ependyma (deeper). 1 – corona radiata; 2 – superior longitudinal fasciculus horizontal segment; 3 – superior longitudinal fasciculus vertical segment; 4 – superior limiting sulcus; 5 – anterior limiting sulcus; 6 – inferior limiting sulcus; 7 – central insular sulcus; 8 – short insular gyri; 9 – long insular gyri; 10 – apex; 11 – sagittal stratum; 12 – extreme capsule; 13 – external capsule; 14 – claustrum; 15 – IFOF; 16 – uncinate fasciculus (UF); 17 – internal capsule; 18 – putamen; 19 – globus pallidus; 20 – lateral ventricle ependyma.

Following the removal of the U-fibers, the morphology of the superior longitudinal fasciculus (SLF), specifically the arcuate fasciculus (AF)—the mesial portion of the SLF—becomes visible (Figure 3). The AF is the first long-distance association pathway identified, forming a C-shaped structure that connects the temporal, occipital, parietal, and frontal lobes while curving around the insula. Topographically, the AF is situated within the depths of the middle frontal gyri, inferior parietal lobule, and superior and middle temporal gyri. Additionally, deep within the temporal segment of the AF lies the inferior longitudinal fasciculus (ILF), positioned inferior and lateral to the temporal horn of the lateral ventricle. The ILF establishes a connection between the anterobasal temporal region and the occipital lobe (Bucheli et al., 2014; Pescatori et al., 2017; Martino and De Lucas, 2014; Martino et al., 2015).

The insular cortex is usually hidden underneath the inner surface of the operculum (Martino et al., 2010). Once the dissection of the cortex and U fibers of the frontal, temporal, parietal, and occipital lobes is completed, it is possible to expose the insular cortex by lifting the operculum (Pescatori et al., 2017; Martino et al., 2015).

To enhance visualization of the white fiber tracts, the resection was extended by removing the insular cortex, followed by the removal of the other insular superficial layers, including the extreme capsule, claustrum, and external capsule. This process exposed the region containing three key structures: the putamen (inferoanterior), the globus pallidus (inferoposterior), and the internal capsule (superior) (Martino et al., 2010).

Dissecting deeper into the occipital lobe reveals the initial fibers of the IFOF. As the dissection follows its trajectory toward the temporal lobe, the fasciculus thickens, while at the frontotemporal transition, it becomes thinner. At this junction, the IFOF is closely associated with the UF, rendering separation between the two fasciculi impossible (Figure 3). Both the IFOF and UF are also identifiable at the ventral portion of the extreme capsule, where their structures narrow significantly. However, the IFOF can be distinguished posterior to the UF in this region (de Benedictis et al., 2021; Pescatori et al., 2017; Martino and De Lucas, 2014; Martino et al., 2015; Martino et al., 2010).

**Figure 4:**
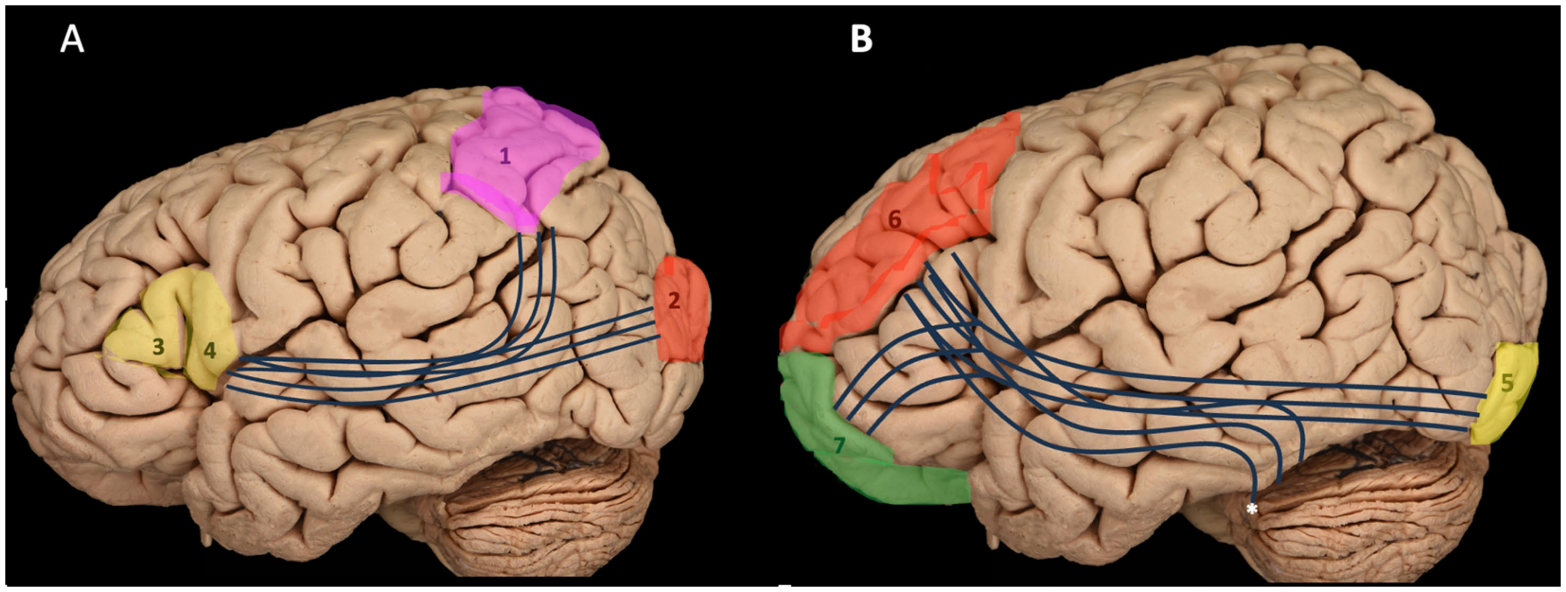
Illustration of both components of the IFOF: the superficial dorsal component **(A)** and the deep ventral component **(B)** with their connections. 1 – superior parietal lobule; 2 – occipital gyrus; 3 – pars triangularis; 4 – pars opercularis; 5 – inferior occipital gyrus; 6 – Middle frontal gyrus and prefrontal cortex; 7 – Frontal pole and orbitofrontal cortex; *, posterior basal temporal region.

This resection demonstrates that the IFOF is an anterior–posterior white matter tract connecting the inferior frontal cortex and dorsolateral prefrontal cortex to the posterior temporal and occipital lobes. It consists of both superficial and deep components (Figure 4) (Tuncer et al., 2021; Wu et al., 2022). The superficial dorsal component links the pars triangularis and pars orbitalis to the superior parietal lobe and the posterior portions of the superior and middle occipital gyri. In contrast, the deep ventral component connects the posterior portion of the inferior occipital gyrus and the posterior basal temporal region to three distinct areas in the middle frontal gyrus (MFG): the dorsolateral prefrontal cortex and the orbitofrontal cortex (Martino and De Lucas, 2014; Martino et al., 2010).

### 3.2 Literature review

#### 3.2.1 Search results

The systematic review identified 510 publications, of which 175 were duplicates across databases. After removing these duplicates, additional reports marked as ineligible by automation tools were excluded. A total of 137 reports were screened, with the majority excluded based on their title or abstract, as they addressed injuries or diseases that did not meet the established criteria. Following this, 110 full-text reports were retrieved for detailed examination. Of these, 88 reports were assessed for eligibility, with most exclusions due to the following reasons: (1) the study described only anatomy, (2) it focused solely on tract connectivity, or (3) it established correlations with white matter fibers outside the study’s scope.

**Figure 5:**
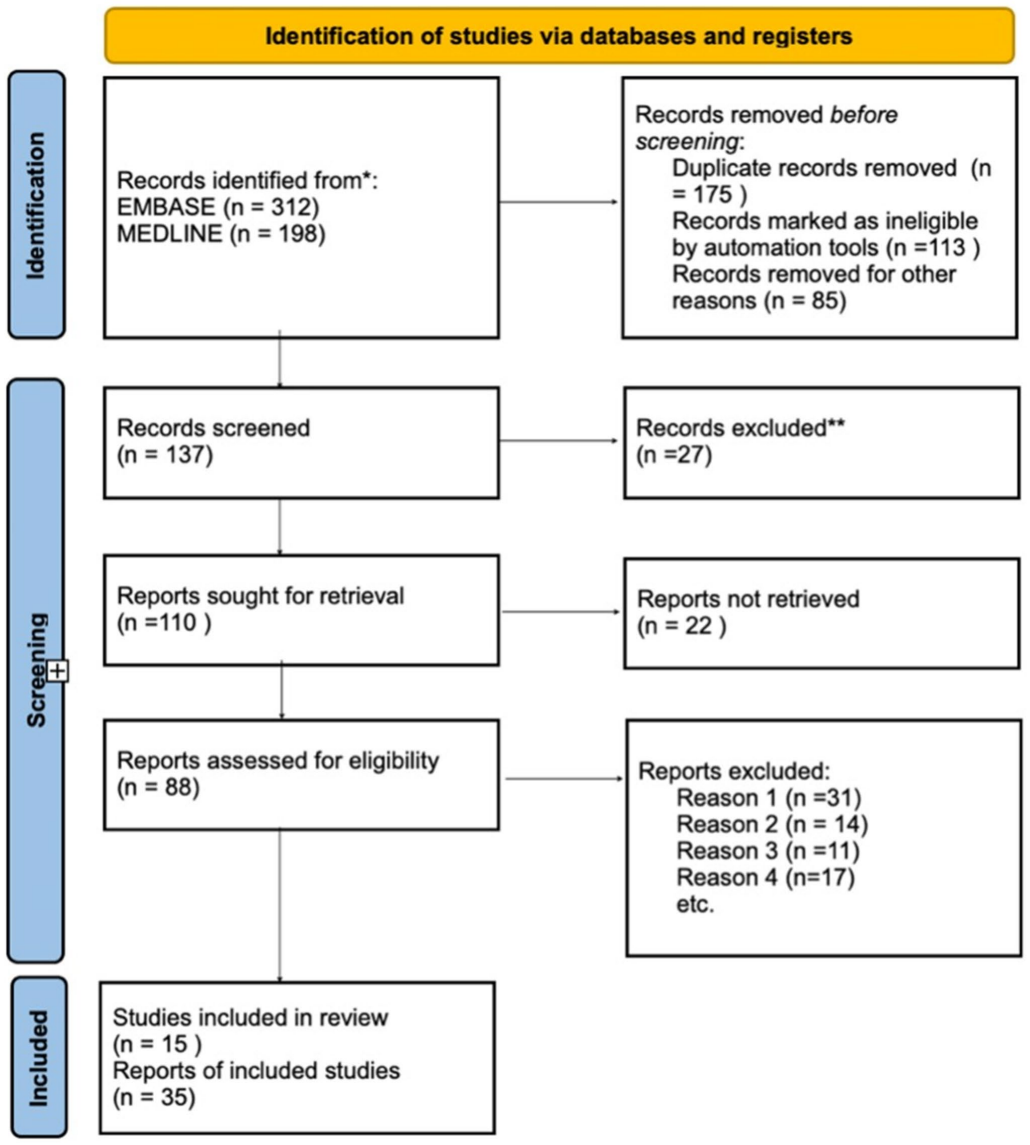
Flowchart illustrating the screening process for article relevancy and eligibility assessment.

Ultimately, 15 publications met the inclusion criteria and were incorporated into the review (Figure 5). These studies consistently highlight that the IFOF is among the longest associative fibers in the human brain, connecting the occipital and frontal lobes. The IFOF plays a key role in the functions of several brain regions, including the auditory, visual, and prefrontal cortices. Its connection with the classical language regions underscores its relevance in contemporary linguistic models, with suggested roles in language comprehension, naming, and reading (Shekari et al., 2021; Tomasino et al., 2024).

#### 3.2.2 Studies analyses

Ille et al. (2018) demonstrated that the IFOF plays a predominant role in language mapping. Among the 10 patients studied, one experienced a permanent surgery-related language deficit. This patient exhibited an overall fiber gain of 49.4%, which was specifically attributed to a distinct increase in IFOF fibers, while other fiber tracts, such as the frontal aslant tract (FAT) and superior longitudinal fasciculus/arcuate fasciculus (SLF/AF), showed minimal changes. Additionally, the study highlights the importance of the IFOF’s anatomical organization in supporting compensatory mechanisms within subcortical structures. This finding is particularly relevant given the greater vulnerability of subcortical white matter pathways compared to cortical regions during surgical resections. This is a critical consideration in glioma patients, as resection-related damage could result in aphasia. Furthermore, the study emphasizes that the combined use of diffusion tensor imaging fiber tracking (DTI-FT) and navigated repetitive transcranial magnetic stimulation (nrTMS) offers promising potential for advancing both research and intraoperative practices.

The IFOF has been shown to mediate semantic language processing by integrating visual information from posterior regions with meaning interpretation in the frontal areas (de Benedictis et al., 2021). Numerous studies analyzing patients with brain lesions within the IFOF’s topography, or simulating IFOF impairment through electrical stimulation, have consistently demonstrated its crucial role in semantic language processing. Lesions or dysfunctions in the IFOF have been increasingly associated with semantic paraphasia, a type of language impairment characterized by the substitution of words with semantically related terms (Chang et al., 2015; Dick et al., 2014; Henderson et al., 2020; Martino and De Lucas, 2014; Martino et al., 2015). This relationship is supported by neuroimaging studies that highlight the IFOF’s role in connecting frontal, temporal, and occipital regions involved in semantic retrieval and processing. Damage to this pathway disrupts the efficient transfer of semantic information, leading to errors in word selection and production, thereby contributing to the manifestation of semantic paraphasia (de Benedictis et al., 2021; Mandonnet, 2017; Duffau, 2012; Cocquyt et al., 2020; Han et al., 2024; Ius et al., 2021).

The IFOF consists of two components: a superficial dorsal segment connecting the frontal lobe to the middle occipital gyrus, and a deep ventral segment linking the frontal lobe to the inferior occipital gyrus. Martino et al. (2010) first identified this distinction in a post-mortem study, revealing that the IFOF could be separated at the ventral portion of the external capsule. This was later confirmed by *in vivo* tractography, which showed that the dorsal IFOF mainly projects to the superior parietal lobule (SPL) in most participants. Caverzasi et al. (2014) used q-ball reconstruction and further confirmed this, identifying frontal cortical connections near the superior and middle frontal gyri. These two pathways are involved in different aspects of linguistic function, and understanding their differential roles is crucial for deciphering the IFOF’s broader contribution to language processing. The ventral pathway of the IFOF is primarily associated with semantic processing, including the integration of auditory, visual, and conceptual information (Chang et al., 2015; Shekari and Nozari, 2023; Dick et al., 2014; Bucheli et al., 2014; Motomura et al., 2014; Eze et al., 2024; Gonzalez Alam et al., 2024; Roux et al., 2021; Nugiel et al., 2016). This pathway facilitates tasks such as word recognition, sentence comprehension, and the processing of meaning in verbal communication. Studies have shown that lesions in the ventral IFOF can lead to impairments in semantic processing, such as difficulties in understanding complex sentence structures and word meaning (Dick et al., 2014). In contrast, the dorsal pathway is more closely related to syntactic processing and the motor aspects of speech production (Chang et al., 2015; Shekari and Nozari, 2023; Dick et al., 2014; Bucheli et al., 2014; Motomura et al., 2014; Eze et al., 2024; Gonzalez Alam et al., 2024; Roux et al., 2021; Nugiel et al., 2016). It supports the integration of linguistic information for syntactic structure, grammar, and speech articulation. Damage to the dorsal pathway can result in deficits in sentence construction and verbal fluency (Shekari and Nozari, 2023) Both pathways, however, are interconnected and work synergistically for effective language comprehension and production, highlighting the IFOF’s role in bridging cognitive and motor aspects of language. Notably, recent studies have emphasized the importance of the IFOF’s dual role in supporting both higher-order cognitive functions (such as semantic processing) and the motoric aspects of language production, reinforcing the idea that the tract operates as a hub within the broader language network (Motomura et al., 2014). Damage to either pathway can result in distinct language deficits, underscoring the complexity of the IFOF’s contributions to communication (Gonzalez Alam et al., 2024; Roux et al., 2021; Nugiel et al., 2016).

Gil-Robles et al. (2013) conducted an electrostimulation study revealing a double dissociation between the ILF and IFOF. Stimulation of the ILF caused impairments in visual object recognition and reading, but not in picture naming. In contrast, stimulation of the IFOF impaired picture naming without affecting visual object recognition or reading. These findings suggest that the IFOF is more involved in semantic processing, while the ILF is linked to visual-orthographic processing (Papagno et al., 2023). While most studies have focused on the IFOF’s role in semantics, recent research has also examined its involvement in reading, phonological, and orthographic processing.

Lesions in the IFOF have been increasingly linked to various cognitive deficits, including prosopagnosia, a condition characterized by the inability to recognize familiar faces despite intact visual perception and memory (Shekari and Nozari, 2023). The IFOF plays a critical role in the integration of visual and social information, being essential for the processing of facial features and the formation of social representations. Dysfunction in the IFOF can impair the ability to access or integrate this information, leading to difficulties in face recognition (Shekari and Nozari, 2023; Young et al., 2021). Recent studies have shown that damage to the IFOF, particularly in its right portion, is strongly associated with deficits in face recognition, especially in contexts requiring the integration of emotional and social information. For instance, a recent study revealed that IFOF lesions can lead to prosopagnosia related to impairments in both visual and emotional pathways, compromising the patient’s ability to recognize faces and interpret facial emotional expressions, which are crucial for social interaction (Shekari and Nozari, 2023). Similarly, another case report observed that patients with IFOF lesions not only had difficulties in face recognition but also exhibited impairments in processing complex visual information, further supporting the idea that the IFOF integrates multiple sensory modalities essential for social and emotional perception (Young et al., 2021) These findings underscore the central role of the IFOF in prosopagnosia, particularly in the visual and emotional processing of faces, suggesting that damage to this neural tract can have significant implications for social recognition and interpersonal communication.

The IFOF, particularly in the right hemisphere, has garnered increasing interest due to its role in both language processing and higher-order cognitive functions. Historically, the IFOF has been implicated in the integration of visual and auditory information, with its left-sided counterpart being crucial for semantic processing and linguistic functions (de Benedictis et al., 2021). However, recent research has expanded our understanding of the right IFOF, suggesting its involvement not only in language but also in social and emotional cognition. Specifically, the right IFOF has been associated with the processing of emotional prosody, the interpretation of non-literal language (e.g., metaphors, humor), and the integration of multimodal sensory information (de Benedictis et al., 2021). Additionally, damage to the right IFOF has been linked to distinct cognitive deficits, such as allocentric neglect, a condition where patients fail to attend to the left side of objects regardless of their spatial orientation. This has been demonstrated in a stroke patient, where allocentric neglect was attributed to injury in the anterior portion of the right IFOF, as revealed through DTI, while no significant damage was observed in other tracts such as the SLF or ILF (Jang and Jang, 2018). This study underscores the importance of the IFOF in attentional processes related to spatial orientation and neglect, highlighting its role in the integration of visual, spatial, and emotional cues essential for proper social communication and understanding. Moreover, these findings suggest that DTI may be a valuable tool in mapping specific brain networks associated with neglect and in elucidating the structural underpinnings of cognitive dysfunctions (de Benedictis et al., 2021; Jang and Jang, 2018).

## 4 Discussion

The anatomy of the IFOF is characterized by its connection between the inferior frontal cortex and dorsolateral prefrontal cortex to the posterior temporal and occipital lobes, comprising both superficial and deep components (Chang et al., 2015; de Benedictis et al., 2021; Shekari and Nozari, 2023; Dick et al., 2014; Mandonnet, 2017; Zhang et al., 2021; Bucheli et al., 2014; Motomura et al., 2014). The dissection of the occipital lobe reveals the initial fibers of the IFOF, which thickens as it follows its trajectory toward the temporal lobe, and it becomes thinner at the frontotemporal junction where it closely associates with the UF, making separation challenging. The superficial dorsal component connects the pars triangularis and pars orbitalis to the superior parietal lobe and the posterior portions of the superior and middle occipital gyri, while the deep ventral component links the posterior inferior occipital gyrus and the posterior basal temporal region to the dorsolateral prefrontal cortex and orbitofrontal cortex in the middle frontal gyrus (Martino and De Lucas, 2014; Martino et al., 2010).

Understanding the anatomy of white matter fibers is critically important in the context of glioma resection, mainly the insulo-opercular tumors, as it significantly influences surgical outcomes and preserves neurological function (Martino et al., 2015; Sanvito et al., 2020). Knowledge of the specific locations and trajectories of key white matter tracts, such as the IFOF and others, enables surgeons to develop strategies that minimize damage to these tracts during tumor excision. Accurate mapping and visualization of these pathways not only assist in maintaining essential cognitive, sensory, and motor functions—such as language and visual processing—but also reduce the risk of postoperative complications (Henderson et al., 2020; Young et al., 2021). Preoperative neuropsychological assessment is crucial for establishing each patient’s baseline cognitive function and guiding surgical planning in neuro-oncology (Ohy et al., 2024). Intraoperatively, awake testing tailored to lesion location - particularly semantic tests like the Pyramid and Palm Tree Test for IFOF involvement - enhances functional preservation strategies (Verst et al., 2021). Ultimately, a thorough comprehension of the anatomy of white matter fibers enhances the surgeon’s ability to achieve a safe and effective resection of gliomas, fostering better patient prognosis and quality of life post-surgery (Henderson et al., 2020; Martino et al., 2015; Young et al., 2021).

IFOF comprises dorsal and ventral components with distinct functional roles in language processing (Henderson et al., 2020). The superficial dorsal component connects frontal regions (pars triangularis and orbitalis) to the superior parietal lobe and posterior occipital gyri, suggesting a primary role in visuospatial processing (de Benedictis et al., 2021; Shekari and Nozari, 2023; Dick et al., 2014; Vandermosten et al., 2012; Stammen et al., 2022). In contrast, the deeper ventral component connects posterior occipital and basal temporal regions (critical for visual object recognition and semantic memory) to the dorsolateral prefrontal and orbitofrontal cortices, indicating a crucial involvement in semantic processing (Chang et al., 2015; de Benedictis et al., 2021; Zhang et al., 2021; Martino and De Lucas, 2014; Martino et al., 2010).

The review emphasizes the dual-pathway model for understanding language development, which highlights the ventral and dorsal streams as key routes for language connectivity (Chang et al., 2015). The UF and IFOF are the primary fiber tracts supporting the ventral stream in language processing. The IFOF plays a crucial role in semantic language processing by integrating visual information from posterior regions with meaning processing in the frontal areas (de Benedictis et al., 2021). Studies involving patients with IFOF lesions or simulated impairments through electrical stimulation consistently show its involvement in semantic processing, often resulting in semantic paraphasia, where conceptually related words are substituted (e.g., tiger for lion) (Chang et al., 2015; de Benedictis et al., 2021; Dick et al., 2014). Additionally, the IFOF supports the integration of sensory-motor, occipital, and frontal areas, facilitating object identification, visual focus, and planning of visually guided movements, mediated by the ventro-lateral prefrontal cortex (de Benedictis et al., 2021).

Beyond language, IFOF lesions have been associated with prosopagnosia, spatial and visual neglect, and impairments in facial emotion recognition (Shekari and Nozari, 2023; Dick et al., 2014; Urbanski et al., 2008). Furthermore, disturbances in picture naming but not in visual object recognition or reading highlight its specific role in semantic and language tasks. Studies on alexic and agraphic patients suggest a connection between the VWFA and frontal semantic regions via the IFOF, supporting its function as a ventral orthographic route (Motomura et al., 2014; Vandermosten et al., 2012). Together, these findings emphasize the multifaceted role of the IFOF in integrating visual, motor, and semantic information.

Highlighting the role of the IFOF in emotional processing is essential, particularly regarding its psychiatric implications. Understanding how IFOF impairments contribute to difficulties in emotion regulation is crucial for recognizing its broader impact on affected individuals. Researchers have shown that white matter alterations significantly impact major depressive disorder (MDD) and bipolar disorder. For instance, Yu et al. (2022) conducted a systematic review on white matter tracts associated with deep brain stimulation targets in MDD, highlighting the involvement of the IFOF among other tracts. Their findings suggest that abnormalities in these tracts may contribute to MDD pathophysiology, potentially affecting emotion regulation by disrupting connectivity between frontal and occipital regions. Additionally, Wei et al. (2020) revealed structural alterations linked to suicide attempts in both MDD and bipolar disorder, which further substantiate the need to understand how disruptions in the IFOF can affect emotional processing in these populations. Overall, these studies highlight the IFOF as a critical tract in the neural circuitry of emotion regulation in both MDD and bipolar disorder. A deeper exploration of this pathway may clarify the neurobiological mechanisms of emotional dysregulation and enhance our understanding of psychiatric condition (Wei et al., 2020).

Tractography and connectivity studies suggest that the IFOF exhibits extensive and variable connections, with evidence of inter-individual differences and hemispheric asymmetries in its cortical terminations (Vassal et al., 2018; Panesar et al., 2017). High-definition fiber tractography (HDFT) provides a more detailed and reproducible view of white matter organization compared to traditional fiber dissection, allowing for improved visualization of large-scale white matter networks. However, while HDFT enhances anatomical mapping, it does not provide direct functional data and has inherent limitations, particularly in resolving crossing fibers, which may lead to overestimation or underestimation of specific connections. Functionally, the IFOF appears to have a layered structure, with its superficial fibers in the dominant hemisphere potentially contributing to language processing, while deeper fibers may be involved in non-language and cognitive functions (Altieri et al., 2019). Given these complexities, further anatomical and functional studies are needed to refine the understanding of the IFOF’s precise role in brain connectivity.

In glioma resection, preserving the integrity of the IFOF is essential for maintaining cognitive functions, particularly language and visual processing. Neurosurgical literature provides key insights into optimizing preoperative planning and intraoperative strategies. Magnetic resonance tractography is a valuable tool for preoperative visualization of the IFOF. It helps identify displaced or disrupted tracts, predicts the presence of eloquent fibers within the tumor, and guides resection planning to minimize postoperative deficits (Li et al., 2023; Sahoo et al., 2023; Mato et al., 2021). Intraoperatively, direct electrical stimulation (DES) maps and preserves functional areas by providing real-time feedback during awake surgery. Integrating DES with tractography enhances the accuracy of identifying eloquent fibers, improving surgical outcomes and reducing functional impairment (Li et al., 2023; Mato et al., 2021; Bello et al., 2008). Careful microsurgical dissection, guided by real-time imaging and DES, further supports language preservation. This multi-modal approach enhances surgical precision, reducing postoperative neurological deficits and improving patient outcomes.

In conclusion, the IFOF plays a pivotal role in integrating visual, motor, and semantic information, making it essential for language and cognitive processing. Anatomically, the IFOF consists of dorsal and ventral components, each with distinct functional contributions. The dorsal component connects frontal areas to parietal and posterior occipital regions, supporting visuospatial and visual-motor integration. In contrast, the ventral component links posterior occipital and basal temporal regions to the frontal cortex, underpinning semantic processing and language functions. Functionally, IFOF disruptions, whether from lesions or electrical stimulation, consistently result in deficits such as semantic paraphasia, spatial neglect, and impaired picture naming, without affecting orthographic processing or visual object recognition. These findings highlight the IFOF’s role in semantic language processing and its broader involvement in tasks such as visual focus, planning visually guided movements, and facial emotion recognition. Integrating anatomical and functional insights into the IFOF can guide surgical strategies, minimize postoperative language deficits, and enhance our understanding of complex cognitive networks.

## Data Availability

The original contributions presented in the study are included in the article/supplementary material, further inquiries can be directed to the corresponding author.
